# Reply to Oleske et al., Blood Lead Concentrations in Newark Children. Comment on “Franklin et al. Broken Promises to the People of Newark: A Historical Review of the Newark Uprising, the Newark Agreements, and Rutgers New Jersey Medical School’s Commitments to Newark. *Int. J. Environ. Res. Public Health* 2021, *18*, 2117”

**DOI:** 10.3390/ijerph18084215

**Published:** 2021-04-16

**Authors:** Rosy C. Franklin, Ryan A. Behmer Hansen, Jean M. Pierce, Diomedes J. Tsitouras, Catherine A. Mazzola

**Affiliations:** 1 New Jersey Pediatric Neuroscience Institute, Morristown, NJ 07960, USA; rozyfranklin2011@gmail.com (R.C.F.); rah229@njms.rutgers.edu (R.A.B.H.); 2 Health Professionals and Allied Employees, Emerson, NJ 07630, USA; jpierce@hpae.org; 3 American Association of University Professors, Biomedical and Health Sciences of New Jersey, Newark, NJ 07103, USA; executive.director@aaupbhsnj.org; 4 Department of Neurosurgery, Rutgers New Jersey Medical School, Newark, NJ 07103, USA

We read, with tremendous gratitude, Dr. Oleske and Dr. Bogden’s comment in the *International Journal of Environmental Research and Public Health* titled, “Blood Lead Concentrations in Newark Children” [1]. We wish to formally thank these authors for emphasizing that Newark’s children, over many decades, have suffered tremendously, and that this is an ongoing issue which warrants attention.

Admittedly, including all pertinent history and data in our review article [2] while staying within scope presented a challenge. Our aim was to briefly describe the relationship between organized medicine, state and local leaders, and the people of Newark. The more information we gathered about Newark’s history, the more systemic injustice we encountered. We found ourselves needing to at least mention complicated topics such as police violence, the COVID-19 pandemic, redlining, and the HIV/AIDS crisis. We firmly believe that these issues all contribute to the many well-known, disparate health outcomes endured by Newark citizens for decades. Nonetheless, we remain acutely aware that our manuscript is by no means a comprehensive review of even Newark’s recent public health history.

Dr. Oleske and Dr. Bogden’s piece adds a critical and necessary point to our discussion. Average pediatric blood lead levels have decreased in Newark over the last forty years, but unacceptable numbers of Newark children are still being poisoned with lead [3]. We particularly appreciate the authors’ connection of blood lead poisoning to the topic of public housing in Newark: as early as 1971, inhalation and/ or ingestion of lead-containing paint chips could be correlated to poorly-maintained and renovated housing in Newark. The issue of lead poisoning is particularly tragic, as it represents a systemic, *generational* injustice: today’s families are still coping with ramifications of past policy decisions. Many communities around the country continue to face a similar egregious, preventable, tragedy [4].

In March 2021, a letter from the New Jersey Department of Health to all health care providers noted, “Throughout the COVID-19 pandemic children have spent significantly more time at home, increasing their risk to exposure of lead paint and contaminated dust” [5]. The letter further describes that, “The number of children with elevated blood lead levels and the number of hospitalizations also increased in 2020” [5]. We must continue to develop evidence-based interventions to mitigate exposure to lead among New Jersey’s most vulnerable populations. This includes Black and Brown children in disadvantaged communities such as Newark.

We appreciate the added discussion by Dr. Oleske and Dr. Bogden and are deeply thankful for these authors’ groundbreaking work to improve the health and wellbeing of children across the world. This includes, but is not limited to, their relentless work to identify and treat children with lead poisoning [6,7,8]. It is time we address these structural inequities at the source. From our perspective, it is time to fulfil the promises made to the people of Newark and, most importantly, to its children.

## References

[1] Oleske J.M., Bogden J.D. (2021). Blood Lead Concentrations in Newark Children. Comment on Franklin, R.C.; Behmer Hansen, R.A.; Pierce, J.M.; Tsitouras, D.J.; Mazzola, C.A. Broken Promises to the People of Newark: A Historical Review of the Newark Uprising, the Newark Agreements, and Rutgers New Jersey Medical School’s Commitments to Newark. Int. J. Environ. Res. Public Health 2021, 18, 2117. Int. J. Environ. Res. Public Health.

[2] Franklin R.C., Behmer Hansen R.A., Pierce J.M., Tsitouras D.J., Mazzola C.A. (2021). Broken promises to the people of newark: A historical review of the newark uprising, the newark agreements, and rutgers new jersey medical school’s commitments to newark. Int. J. Environ. Res. Public Health.

[3] Childhood Lead Exposure in New Jersey Annual Report New Jersey Department of Health. https://www.state.nj.us/health/childhoodlead/documents/reports/childhoodlead2019.pdf.

[4] Pell M.B., Schneyer J. (2017). Off the Charts: The thousands of U.S. locales where lead poisoning is worse than in Flint. Reuters Investigates.

[5] Childhood Lead Provider Letter New Jersey Department of Health. https://www.state.nj.us/health/childhoodlead/documents/2021_ChildhoodLead_provider%20letter.pdf.

[6] Shah K.K., Oleske J.M., Gomez H.F., Davidow A.L., Bogden J.D. (2017). Blood lead concentrations of children in the united states: A comparison of states using two very large databases. J. Pediatr..

[7] Gómez H.F., Borgialli D.A., Sharman M., Weber A.T., Scolpino A.J., Oleske J.M., Bogden J.D. (2019). Blood lead levels in females of childbearing age in flint, michigan, and the water crisis. Obstetr. Gynecol..

[8] Gómez H.F., Borgialli D.A., Sharman M., Shah K.K., Scolpino A.J., Oleske J.M., Bogden J.D. (2018). Blood lead levels of children in flint, michigan: 2006–2016. J. Pediatr..

